# Unveiling the dynamic coupling and driving mechanisms among digital infrastructure, economic resilience and carbon productivity

**DOI:** 10.1371/journal.pone.0333309

**Published:** 2025-10-27

**Authors:** Jie Huang, Beibei Zheng, Narisu Liu, Xi Chen

**Affiliations:** 1 School of Business, Xinyang Normal University, Xinyang, China; 2 Research Institute of the Economic and Social Development in the Dabie Mountains, Xinyang Normal University, Xinyang, China; 3 School of Economics and Management, Jiaying University, Meizhou, China; SRM University AP, INDIA

## Abstract

Investigating the coupled and coordinated relationship among digital infrastructure (DI), economic resilience (ER) and carbon productivity (CP) is pivotal for advancing sustainable development in China. This study employs multiple approaches, including the coupling coordination model, kernel density estimation, Markov chain, and spatial Durbin model, to analyze the spatiotemporal evolution and driving factors of the coupling coordination degree (CCD) from 2013 to 2021. The findings are as follows. First, the average levels of DI, ER and CP show a consistent upward trajectory. Second, the CCD among the three systems exhibits steady growth, transitioning from a state of “general out of balance” to “barely coupling coordination”. Spatially, the CCD demonstrates a characteristic pattern of decreasing from east to west. Overall, achieving a “cross-level transition” in CCD is challenging, and the neighboring provinces significantly influence the enhancement of local CCD. Third, the CCD demonstrates a positive spatial agglomeration effect, with stable hotspots concentrated in regions such as Hubei, Anhui, Zhejiang, and Shanghai. Fourth, strategic emerging industries, population density, human capital, and environmental regulation intensity positively contribute to the local CCD, whereas financial development exerts a negative impact. Regarding spatial spillover effects, strategic emerging industries and environmental regulation intensity exert positive influences, while population density shows a negative effect. Meanwhile, the spatial spillover effects of human capital and financial development are not significant.

## 1 Introduction

As global climate change intensifies, ecosystems and economic systems face severe challenges. Without effective carbon emission control, global warming could exceed 1.5 °C by mid-century, triggering irreversible ecological disruptions [1]. Meanwhile, the World Economic Forum (WEF) *Global Risks Report 2023* identifies climate-related risks as persistent threats to global economic stability. Intensifying climate shocks reveal the vulnerability of economic systems [2], highlighting the urgent need to enhance economic resilience (ER) while reducing emissions. Improving carbon productivity (CP) is critical to achieving this balance, yet meeting 2050 climate targets requires a nearly tenfold increase in CP [3,4]. Under dual pressures of low-carbon transition and economic security, digital infrastructure (DI) plays a growing role in green growth. By enhancing energy efficiency, environmental monitoring, and system resilience, DI supports both emission reduction and economic development [5,6]. National strategies such as the *14th Five-Year Plan for Digital Economy Development* and the Digital China initiative have accelerated DI deployment and digital advancement. Through these policies, DI has emerged as a new economic growth engine and a driver of ER and CP. In this context, clarifying the coupling coordination relationships among DI, ER, and CP is of great practical significance for promoting regional sustainable development.

At present, most domestic and international studies can be broadly categorized into three strands. First, regarding the relationship between DI and CP, some scholars argue that DI enhances CP by promoting technological innovation [7], upgrading industrial structures [8], and green transformation of residents’ lifestyles [9]. In addition, the positive effect of DI on CP is not confined to the local areas. Several studies have demonstrated that it also exerts positive spatial spillover effects on surrounding regions [10,11]. However, other researchers have highlighted a potential “double-edged sword” effect of DI. According to Feng et al. [12], based on firm-level data, the expansion of DI may increase carbon intensity and reduce the CP of small enterprises through competitive pressures and changes in energy consumption structures. Some scholars suggest that the construction and operation of DI can directly increase energy consumption and diminish the efficiency of the digital economy, thereby reducing urban CP [13–15]. In summary, existing studies have primarily examined the impact of DI on CP, yet the direction of its effects remain unclear.

Second, existing studies on the direct relationship between ER and CP remain limited, with most research focusing on the link between ER and carbon emissions. These studies generally find that improving environmental performance contributes to enhancing ER. Shi et al. [16] pointed out that emission reduction measures, including the promotion of a circular economy, technological progress, and industrial structure adjustment, can enhance ER. Zhao and Jiang [17] found that lowing carbon intensity significantly boosts ER. Enhancing ER, in turn, supports green transitions and emission reduction by fostering economic diversification and reducing dependence on carbon-intensive industries [18,19]. Nevertheless, some scholars argue that the excessive pursuit of ER may lead to overinvestment in critical infrastructure, increasing emissions [20]. Third, the relationship between DI and ER has attracted growing academic attention [21]. Particularly during the COVID-19 pandemic, studies showed that well-developed digital infrastructure enhanced regional resilience to external shocks [22,23]. Empirical evidence based on difference-in-differences models confirms that DI strengthens urban ER [24] and generates positive spatial spillover effects [25].

Building on the above analysis, prior studies has primarily focused on bilateral relationships among DI, ER, and CP, while neglecting their integrated interactions and systemic coordination. As DI, ER, and CP are not independent subsystems, their mutual interactions form internal linkages. Therefore, fostering a virtuous cycle and achieving synergy among the three systems is essential for enhancing the overall efficiency and stability of socio-economic-environmental systems. In response to these gaps, this study selects 31 provinces in China from 2013 to 2021 as the empirical sample. It applies the global entropy method to assess the development levels of DI and ER, and adopts the coupling coordination degree (CCD) model to assess the interactive relations among DI, ER and CP. To capture spatial-temporal dynamics, this study integrates kernel density estimation, Markov chains, and spatial autocorrelation to reveal the distribution, evolution, and clustering of CCD. The SDM is further applied to assess spillover effects and identify key drivers. This multi-dimensional approach helps deepen comprehension of the interaction mechanisms among DI, ER and CP. It also provides theoretical and policy guidance for advancing sustainable regional development.

This research offers the following marginal contributions. First, it constructs an integrated analytical framework to examine the spatiotemporal coupling coordination among DI, ER, and CP, addressing the lack of holistic perspectives in existing research that mostly focuses on pairwise interactions [16,21,26,27]. Second, it investigates how influencing factors affect CCD in neighboring regions via spatial spillovers. By incorporating the SDM, the study captures both local and spillover effects, enriching understanding of the pathways toward synchronized development among DI, ER and CP across cities. Third, it employs kernel density estimation, Markov chains, and trend surface analysis to reveal the distributional dynamics, transition probability, and spatial evolution of CCD, providing comprehensive insights into its spatiotemporal evolution.

The subsequent sections are arranged as follows. Section 2 outlines the materials and methods. Section 3 displays the study results. Section 4 discusses the findings in detail. Section 5 concludes the study. Section 6 offers policy recommendations.

## 2 Materials and methods

### 2.1 Research framework

**Fig 1:**
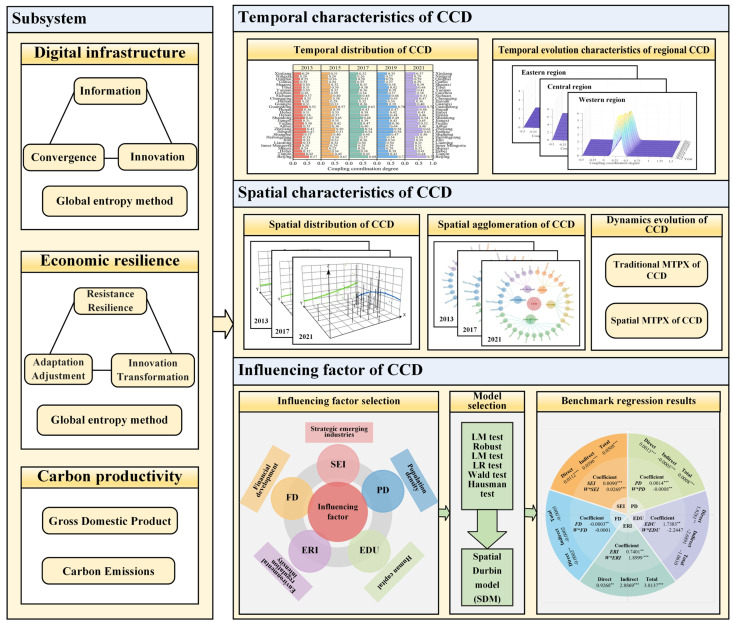
Analysis framework.

To explore the coupling and coordination relationship between DI, ER and CP in China, this research is conducted in four steps (Fig 1). Firstly, this paper elucidates the interaction mechanism between DI, ER and CP. Secondly, an index system for DI, ER and CP is established, and the levels of the three systems in China are measured. Thirdly, using panel data for 31 provincial administrative areas between 2013 and 2021, a CCD model is developed using the linear assignment method. The harmonious development of the three systems is quantitatively analyzed across temporal and spatial dimensions. Finally, spatial autocorrelation is employed to evaluate the relationships among three systems. Based on this analysis, the SDM is employed to investigate the factors influencing the CCD. Subsequently, relevant policy recommendations are proposed, reflecting the coordinated development status across provinces, to provide strategic paths for enhancing the sustainability of these three systems.

### 2.2 Coupling mechanism

DI, ER and CP are key drivers of green and low-carbon development [28]. These three systems are interconnected and mutually reinforcing, making it crucial to explore their relationships for the sustainable development.

The progress of infrastructure systems is significant for every stage of economic and social development in China [29]. DI significantly contributes to promoting sustainable economic development [30]. On the one hand, the data stored in digital infrastructures can exert a “leverage effect”, effectively utilizing limited resources and strengthening the resistance and adaptability of economic systems. On the other hand, the information platforms provided by DI promote the agglomeration of talent and capital, thus upgrading the innovation potential of the economic system [30]. DI also positively impacts green innovation [31]. Progress in DI accelerates technological innovation, enhances productivity, and reduces energy losses, resulting in positive effects on CP [32]. Concurrently, the development of information infrastructure diminishes market information asymmetry and improves resource allocation efficiency, consequently increasing CP [33,34].

ER creates favorable conditions for the growth of DI. Compared to traditional infrastructure, the technological structure of DI, which embodies intelligent features, is more complex. A resilient economic system mitigates unanticipated economic shocks and ensures the economic security of DI. The strategic objective of establishing a resilient economic system also guides the practical development of DI, ensuring that it meets the requirements of sustainable, intelligent, and efficient operation. Additionally, ER is crucial for the advancement of a low-carbon economy [16]. Resilient economic systems are better able to adapt to changes in environmental policies, thereby facilitating the deployment of low-carbon technologies and renewable energy sources. Improving urban ER can also facilitate enterprise transformation, reduce resource consumption, and enhance CP through industrial structure upgrading [35].

Low carbon economic development offers strategic direction for DI. The “14th Five-Year Plan for Green Industrial Development” requires phasing out high-energy-consuming and low-efficiency enterprises, thereby freeing up resources and space to foster low-carbon industries and accelerating the development of DI. Additionally, ecological improvement is a crucial guarantee for stable economic development. As pollution problems worsen, the government has begun to implement stringent environmental standards and market requirements, compelling enterprises to proactively innovate in technology to maintain sustainable regional economic development. Moreover, in alignment with green development goals, China is striving to improve energy efficiency in energy-intensive sectors, including 5G and data centers, through technological advancements and innovation. This approach guides science and technology towards a green economy. Research has also shown that regional ecological environments significantly influence talent attraction. Enhancing the ecological environment draws in high-quality and highly-skilled talent [36], which effectively enhances the regional technology level.

### 2.3 Indicator system

#### 2.3.1 Evaluation indicators system of digital infrastructure.

The National Development and Reform Commission (NDRC) explicitly outlined DI on April 20, 2020, comprising three components: information, convergence, and innovation infrastructure. Information infrastructure describes the hardware and software systems used to support the transmission, storage and processing of information. Convergence infrastructure describes facilities that combine traditional infrastructure with digital technologies to enable intelligent and efficient management. Convergence infrastructure is measured by the development stage of traditional infrastructure and the information industry. Innovation infrastructure comprises facilities and platforms that support scientific research, technological advancement, and industrial innovation. Building upon previous studies, this paper selects relevant indicators to evaluate the level of DI across three dimensions: information, convergence, and innovation infrastructure [37–39], as summarized in Table 1. The weights assigned to the DI indicators were calculated using the global entropy method, which provides an objective and data-driven approach based on variability across regions and time periods. Unlike the conventional entropy method applied to single-year data, the global entropy method accounts for both cross-sectional and temporal variation. This enables a more comprehensive evaluation of each indicator’s informational contribution and produces more stable and representative weights for longitudinal analysis. By reducing subjectivity, the method improves the methodological rigor and enhances the comparability of composite indices over time.

**Table 1:** Evaluation indicators system of DI.

System	Dimension		Indicator	Type	Weight
Digital infrastructure	Information infrastructure		Number of domain names (ten thousand)	+	0.034
			Number of web pages (ten thousand)	+	0.054
			Internet broadband access port (ten thousand)	+	0.016
			Mobile internet users (10,000 households)	+	0.015
			Number of computers in use at the end of the period (unit)	+	0.027
			Computers per 100 population (unit)	+	0.013
			Number of websites owned by enterprises (number)	+	0.026
			Number of enterprises with e-commerce trading activities (number)	+	0.028
			Local exchange capacity (10,000 households)	+	0.027
			Mobile switch capacity (10,000 households)	+	0.014
			Cell phone base station (ten thousand)	+	0.015
			Fiber optic line length(km)	+	0.017
			Cell phone penetration rate (departments/100 people)	+	0.008
			Average population served per outlet for postal communications (10,000 people)	+	0.011
			Total length of cable radio and television transmission trunk networks (10,000 km)	+	0.027
			Actual number of cable broadcast television subscribers (10,000 households)	+	0.014
			Postal business outlets (division)	+	0.018
			Mailboxes (number)	+	0.023
			Rural delivery routes (km)	+	0.013
			City delivery routes (km)	+	0.013
			Total length of postal routes (km)	+	0.024
	Convergence infrastructure	Traditional infrastructure	Railroad mileage (km)	+	0.012
			Miles of high-speed graded roads (km)	+	0.011
			Total length of routes operated by public trolley buses (km)	+	0.022
			Miles of rail transit in operation (km)	+	0.042
			Public buses per 10,000 population (unit)	+	0.006
			Health care institutions (number)	+	0.018
			Number of general higher education institutions (number)	+	0.056
			Public library (number)	+	0.012
			Public library holdings per capita (volume)	+	0.020
			Electricity consumption by region (billion kw·h)	+	0.016
		Level of development of the information industry	Revenue from software operations (billions)	+	0.046
			Enterprise e-commerce sales (billions)	+	0.035
			Enterprise e-commerce purchases (billions)	+	0.038
			Total postal and telecommunication operations (billions)	+	0.031
			Total telecommunication services (billions)	+	0.031
	Innovation infrastructure		Full-time equivalent of R&D personnel (person-years)	+	0.029
			R&D funding (ten thousand yuan)	+	0.030
			Number of R&D projects (item)	+	0.039
			Number of patent applications (piece)	+	0.033
			Number of patents granted (piece)	+	0.036

#### 2.3.2 Evaluation indicators system of economic resilience.

Martin [40] proposes four dimensions of regional ER: the ability to reorganize and innovate after an economic shock, the resilience to withstand shocks, the ability to internally integrate and adapt to new external environments when impacted by a shock, and the ability to recover from a shock. Given the strong correlation between resistance and resilience, which makes them difficult to separate at the data level, this paper constructs the indicator system based on resistance and resilience, adaptation and adjustment, and innovation and transformation. The specific content is detailed in Table 2. The weights assigned to the indicators of ER was calculated using the global entropy method.

**Table 2:** Evaluation indicators system of ER.

System	Dimension	Indicator	Type	Weight
Economic resilience	Resistance and resilience	Per capita GDP (yuan)	+	0.068
		Per capita disposable income of urban residents (yuan)	+	0.061
		Urban registered unemployment rate (%)	–	0.012
		Unemployment insurance participation rate (%)	+	0.097
	Adaptation and adjustment	Local general budget expenditure per capita (yuan/person)	+	0.088
		Employees over total regional population (%)	+	0.031
		Total general budget fiscal expenditures over total revenues (%)	+	0.115
		Urbanization rate (%)	+	0.018
	Innovation and transformation	Tertiary sector as a share of GDP (%)	+	0.036
		Science and technology expenditures as a share of GDP (%)	+	0.082
		Level of foreign direct investment (%)	+	0.110
		Number of patents granted for inventions (piece)	+	0.212
		Financial expenditure on education (billions)	+	0.069

#### 2.3.3 Evaluation indicators system of carbon productivity.

CP quantifies the economic output generated for each unit of carbon emissions, acting as a key indicator for low-carbon development [41]. This paper acquired the latest panel data on carbon dioxide emissions from EDGAR covering 2013–2021. The CP formula is:


$${Cp}_{it}=\frac{{GDP}_{it}}{{{CO}_{\text{2}}}_{it}}$$

where ${GDP}_{it}$ and ${{CO}_{\text{2}}}_{it}$ denote the Gross Domestic Product and carbon emissions, respectively, of city t in period i.

### 2.4 Methods

#### 2.4.1 Global entropy method.

If we evaluate p variables in n regions over t years, we can collect data to get t cross-sectional data tables $X^{t}={\left(X_{ij}\right)}_{n\times p}$, and introduce the global idea to arrange these t tables in chronological order from top to bottom, resulting in a global evaluation matrix of size nt×p, which is written as $X={\left(X^{\text{1}},X^{\text{2}},\cdot \cdot \cdot ,X^{t}\right)}_{nt\times p}$.

Because the data in the global evaluation matrix X are quite different in terms of scale, unit of measurement, order of magnitude, etc., they cannot be synthesized and calculated directly, and they must be normalized. The standardization process is defined as follows:


$$\text{Negative indicators}:\;{x^{\prime }}_{ij}=\frac{X_{ij}-minX_{ij}}{maxX_{ij}-minX_{ij}}\times \text{0}.\text{9}\text{9}+\text{0}.\text{0}\text{1}$$ (1)

$$\text{Positive indicators}:\;{x^{\prime }}_{ij}=\frac{maxX_{ij}-X_{ij}}{maxX_{ij}-minX_{ij}}\times \text{0}.\text{9}\text{9}+\text{0}.\text{0}\text{1}$$ (2)

$$f_{ij}(t)=\frac{{x^{\prime }}_{ij}}{\sum_{j=\text{1}}^{nt}{x^{\prime }}_{ij}}(\text{1}\leq i\leq nt,\text{1}\leq j\leq p)$$ (3)

$$\begin{array}{c} e_{j}=-\frac{\text{1}}{\text{ln}nt}\sum_{i=\text{1}}^{nt}f_{ij}\times \text{ln}\;f_{ij}(\text{1}\leq i\leq nt,\text{1}\leq j\leq p) \end{array}$$ (4)

$$g_{j}=\text{1}-e_{j}$$ (5)

$$w_{j}=\frac{g_{j}}{\sum_{j=\text{1}}^{p}g_{j}}$$ (6)

$$\begin{array}{c} Z=\sum_{j=\text{1}}^{p}w_{j}{x^{\prime }}_{ij} \end{array}$$ (7)

where, ${x^{\prime }}_{ij}$ is the standardized indicator value, $X_{ij}$ denotes the original value of the j indicator in the i region. $f_{ij}(t)$ denotes the proportion of region i for indicator j. $e_{j}$ denotes the information entropy of indicator j. $g_{j}$ denotes the coefficient of variation for indicator j. $w_{j}$ denotes the weight of indicator j. Z denotes the comprehensive index.

#### 2.4.2 Coupling coordination degree model.

The CCD measures how well these systems interact and coordinate with each other [42].

Equation for the coupling of the three systems:


$$\begin{array}{c} CD=\sqrt[{\text{3}}]{\frac{U_{\text{1}}\times U_{\text{2}}\times U_{\text{3}}}{({\frac{U_{\text{1}}+U_{\text{2}}+U_{\text{3}}}{\text{3}})}^{\text{3}}}} \end{array}$$ (8)

where, U denotes the comprehensive score of the i subsystem, respectively. CD is the degree of three-system coupling.

The CCD model of the three systems is constructed as follows:


$$CCD=\sqrt{CD\times T}$$ (9)

$$T=\alpha_{\text{1}}U_{\text{1}}+\alpha_{\text{2}}U_{\text{2}}+\alpha_{\text{3}}U_{\text{3}}$$ (10)

where, T represents the total comprehensive index of the three systems, and α represents the weight assigned to each subsystem, reflecting the importance of the three systems, and equal importance is assumed in this paper, so the three coefficients are all set to 1/3. According to the previous research [42], CCD is categorized as follows.

#### 2.4.3 Kernel density estimation.

The kernel density estimation (KDE) can examine the trend of spatiotemporal variation of the estimated samples by constructing the density function and based on the shape of the estimated distribution. The kernel density function is defined as follows:


$$\begin{array}{c} f(x)=\frac{\text{1}}{Nh}\sum_{i=\text{1}}^{N}K(\frac{x_{i}-\overset{―}{x}}{h}) \end{array}$$ (11)

$$K(x)=\frac{\text{1}}{\sqrt{\text{2}\pi }}exp\left(-\frac{x^{\text{2}}}{\text{2}}\right)$$ (12)

where, x is a random variable, N is the number of provinces, $x_{i}$ denotes the i province composite index, $\overset{―}{x}$ represents the average composite index value. K(x) denotes the KDE, h represents bandwidth, which is used to determine the accuracy in the KDE, in general, a smaller bandwidth value increases estimation accuracy but results in a less smooth curve.

#### 2.4.4 Markov chain analysis.

Traditional Markov chain analysis. It analyzes the probability of a region’s three-system CCD transitioning to either a lower or higher level through the construction of a state transition matrix. The Markov chain is represented as a stochastic process$\;(\{X_{t},\;t\in T\})$, where T represents different time periods, while the finite states refer to the number of possible states of the random variable. If the three-system CCD is divided into k levels, a k×k transition probability matrix is obtained. This matrix illustrates the dynamic evolution trend of the three-system CCD in China by analyzing state transition probabilities.

Spatial Markov chain analysis. To assess the impact of spatial factors on state transition probabilities, Whittaker and Thomason [43] constructed a spatial Markov chain. First, a spatial weight matrix is established. Then, the traditional k×k transition probability matrix is decomposed into k conditional k×k transition probability matrices, incorporating different spatial lag types k. Under the condition k, the spatial transition probability from type i to type j at time t is analyzed. This approach reveals the effect of spatial factors on the transition trends of CCD.

#### 2.4.5 Global spatial autocorrelation.

The level of CCD among DI, ER and CP may be spatially autocorrelated, and the global Moran’s I is measured with the following formula:


$$Moran^{\prime }s\;I=\frac{n\sum_{i=\text{1}}^{n}\sum_{j=\text{1}}^{n}w_{ij}\left(x_{i}-\overset{―}{x}\right)\left(x_{j}-\overset{―}{x}\right)}{\sum_{i=\text{1}}^{n}\sum_{j=\text{1}}^{n}w_{ij}\sum_{i=\text{1}}^{n}{\left(x_{i}-\overset{―}{x}\right)}^{\text{2}}}$$ (13)

where, n is the sample size, $x_{i}$ and $x_{j}$ are the spatial unit observations of i and j. $\overset{―}{x}$ denotes the mean of spatial observations. $w_{ij}$ denotes the domain relationship between spatial units i and j, equal to 1 when i and j are adjacent, and 0 otherwise.

#### 2.4.6 Spatial measurement model.

To test the spatial dependence of CCD and to estimate the influencing factors, this paper employs the SDM model. The model is detailed below:


$$\begin{array}{c} Y_{it}=\beta_{\text{0}}+\rho \sum_{j=\text{1}}^{n}w_{ij}\times Y_{jt}+\beta_{\text{1}}X_{it}+\rho_{\text{1}}\sum_{j=\text{1}}^{n}w_{ij}\times X_{jt}+\mu_{t}+\lambda_{i}+\varepsilon_{it} \end{array}$$ (14)

where $Y_{it}$ is the CCD of province i in year t. ρ is the spatial autoregressive coefficient of CCD, and $\rho_{\text{1}}$ captures the spatial spillover effects of the explanatory variables. $\beta_{\text{0}}$ is the constant term, and $\beta_{\text{1}}$ is the coefficient of the local explanatory variables. $X_{it}$ and $X_{jt}$ denote the local and neighboring explanatory variables, respectively. $w_{ij}$ is the spatial weight matrix, specified as a 0–1 matrix where $w_{ij}=\text{1}$ if provinces i and j share a border, and 0 otherwise. $\mu_{t}$ and $\lambda_{i}$ represent time and individual fixed effects, respectively. $\varepsilon_{it}$ is the random error term.

### 2.5 Data resources

The observation sample in this research consists of 31 provincial-level administrative units. Following Nie et al. [44] classifications, it is categorized into three regions: eastern, central and western. Considering the data availability and timeliness, the study period spans from 2013 to 2021. The study data are from China Statistical Yearbook, China Science and Technology Statistics Yearbook, China Information Industry Yearbook and EDGAR, a Global Database of Atmospheric Emissions.

## 3 Results

### 3.1 The comprehensive evaluation index of three systems

#### 3.1.1 From the national perspective.

**Fig 2:**
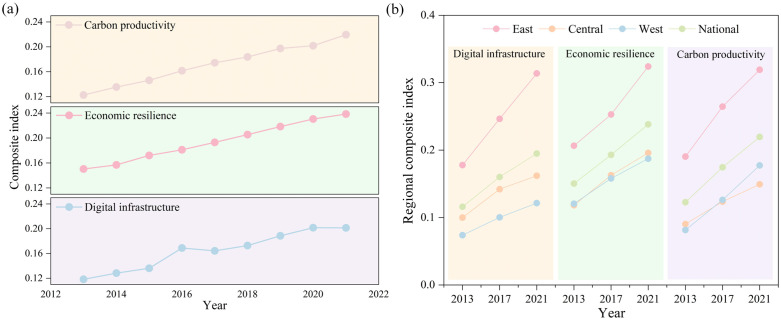
Comprehensive index of the DI, ER and CP. (a) Mean levels of DI, ER and CP. (b) Regional composite index of DI, ER and CP.

Fig 2a illustrates the trend in the average values of DI, ER and CP. Overall, the national DI composite index demonstrates a dynamic upward trend, rising from 0.118 in 2013 to 0.201 in 2021, showing a yearly average rise of 6.88%. The comprehensive index of ER increased from 0.150 to 0.238 during the same period, growing at an average rate of 5.92% per year. Similarly, the national CP rose steadily from 0.123 to 0.220, corresponding to an average annual increase of 7.55%. This suggests that the ER level exceeds that of DI and CP. However, the growth rates of DI and CP are higher than of ER.

#### 3.1.2 From the regional perspective.

The comprehensive indices of DI, ER and CP across regions are illustrated in Fig 2b. Regionally, the eastern area consistently leads in DI, significantly exceeding the national average, while the central and western regions remain below it. In 2013, the DI index exhibited a clear regional hierarchy, with the eastern region (0.178) ranking highest, followed by the national average (0.116), the western region (0.074), and the central region (0.010). By 2021, while the overall ranking remained consistent, all regions experienced notable improvements. The eastern region maintained a significant lead (0.314), while both the central (0.162) and western (0.102) regions showed marked progress, although they continued to lag behind the national average (0.160). This reflects the pronounced regional disparities in the level of DI.

ER levels have shown a steady upward trend nationwide, with the eastern region maintaining its leading position and accelerating in recent years. Conversely, the central and western regions have remained stable and below the national average. In 2013, ER rankings were as follows: eastern (0.206), national average (0.150), western (0.120), and central (0.118). By 2021, the eastern region still ranked highest (0.324), while the central region (0.161) slightly surpassed the western region (0.157); the national average rose to 0.238. Prior to 2015, the western region consistently outperformed the central region in ER, but this trend reversed thereafter, reflecting faster progress in the central region.

CP levels also exhibited significant regional variation. The eastern region maintained an advantage over the national average throughout the period, while the central and western regions lagged behind. In 2013, CP values ranked as follows: eastern (0.190), national average (0.123), central (0.090), and western (0.081). By 2021, although the eastern region remained dominant (0.319), the western region (0.177) overtook the central region (0.149); the national average rose to 0.219. Notably, before 2017, the central region consistently recorded higher CP levels than the western region, a trend that was reversed in subsequent years, indicating relatively accelerated growth in the west.

### 3.2 The spatiotemporal evolution of CCD

#### 3.2.1 The temporal evolution of CCD.

**Fig 3:**
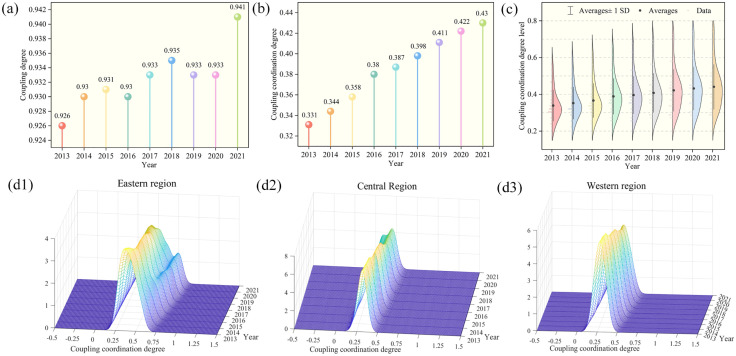
Temporal evolution of CCD. (a) National average of coupling degree. (b) National average of CCD. (c) Time-series distribution of CCD. (d) Kernel density results for CCD among eastern (d1), central (d2), and western regions (d3).

Using the comprehensive indices of DI, ER and CP, and applying equations (8)–(10), the average coupling degree and CCD for the three systems were calculated. As shown in Fig 3a and (b), the average national levels of coupling degree and CCD showed varying degrees of fluctuation and increase. The coupling degree increased from 0.926 to 0.941, reflecting a yearly average rise of 0.20%, while the CCD climbed from 0.331 to 0.430, showing a yearly average rise of 3.32%. The higher coupling degree value indicates a strong degree of interdependence and mutual influence among the subsystems. Although the CCD value is lower, its development trend is favorable. According to the CCD grade judgment criteria in Table 3, the state was generally out of balance from 2013 to 2018, transitioning to a barely coupling coordination state from 2019 to 2021. This indicates that the CCD among the three systems is gradually shifting from a disordered state to a coordinated state. With the gradual increase in the levels of DI, ER and CP, the three systems are expected to achieve higher levels of coordination in the coming years.

**Table 3:** Evaluation standards of the CCD.

Coupling coordination	Type of coordination	Category of levels
0.000 ≤ CCD < 0.200	Extremely out of balance	Ⅰ
0.201 ≤ CCD < 0.400	General out of balance	Ⅱ
0.401 ≤ CCD < 0.500	Barely coupling coordination	Ⅲ
0.501 ≤ CCD < 0.600	Primary coupling coordination	Ⅳ
0.601 ≤ CCD < 0.800	Good coupling coordination	Ⅴ
0.801 ≤ CCD < 1.000	High-quality coupling coordination	Ⅵ

Fig 3c shows the trend in the time-series distribution of CCD in the three systems over the study period. The distribution curve generally shifts upwards year by year, indicating continuous improvement in the CCD and a noticeable increase in provinces achieving coordinated. Regarding the distribution pattern, the main peak of the curve exhibits a broadening trend in width, accompanied by a diminishing trend in height. In 2013, the majority of national CCD levels clustered around 0.3, whereas by 2021, most of them are concentrated around 0.3–0.5. This shift indicates that the CCD levels are gradually becoming discrete, and the spatial differences are more obvious, but the CCD levels of the provinces are on the rise. From the perspective of distribution extensibility, the upward shift feature is evident, with certain provinces and regions exhibiting CCD values that deviate from the national average. For example, Beijing and Guangdong had CCD values exceeding 0.7 in 2021, which is markedly higher than the national average level of 0.43.

This research utilizes the KDE method to analyze the temporal evolution of CCD among DI, ER and CP, illustrated in Fig 3d. The evolution of CCD distribution for these three systems in the eastern region reveals the following: the KDE curve exhibits a rightward shift in its center, signifying a steady enhancement in the CCD for the eastern region. Regarding the distribution pattern, the peak height of the KDE curve displays a downward trend, while the peak’s width expands, indicating an increasing dispersion in CCD across the eastern region. Concerning the distribution ductility, the KDE curve does not exhibit an obvious tail dragging phenomenon, which demonstrates that the CCD among provinces in the eastern regions does not show extremely high or low values. From the perspective of polarization trends, the KDE curve has gradually evolved from a single peak to double-peaked pattern, indicating a significant increase in polarization trends in the eastern regions. This points to a clear gradient effect and widening differences in intra-regional development.

In the central region, the KDE curve exhibits a rightward shift in its center, reflecting continuous improvement in CCD levels. Regarding the distribution pattern, the KDE curve’s peak has gone through a change from “sharp and narrow” to “flat and broad”, signifying that the CCD distribution in the central region has shifted from high concentration to greater decentralization. Concerning the distribution ductility, the KDE curve does not exhibit an obvious tail dragging phenomenon, which demonstrates that CCD differences among central provinces are not expanding. From a polarization perspective, the KDE curve has transitioned from a single peak to a pattern with both a primary and secondary peak, reflecting an increase in the number of peaks. This suggests that the CCD within the central regions is exhibiting a trend of polarization.

The CCD distribution in the western region has gradually shifted rightward on the KDE curve, reflecting steady improvement in CCD levels over time. In terms of distribution, the peak height of the KDE curve shows a slight decline, suggesting an emerging—but still limited—increase in internal disparity. However, the KDE curve remains single-peaked, and no significant long-tail pattern is observed, implying that most provinces are still clustered around similar CCD levels. From a polarization perspective, this reflects a unipolar distribution, with relatively moderate internal variation and a tendency toward spatial convergence. These provinces are transitioning from a state of general out of balance to barely coupling coordination.

#### 3.2.2 The spatial distribution of CCD.

**Fig 4:**
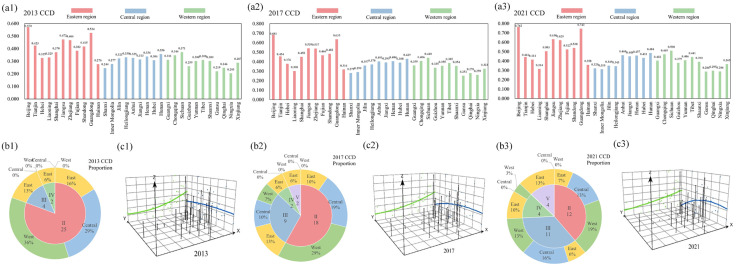
Spatial distribution of CCD. (a) Regional distribution of CCD in 2013 (a1), 2017 (a2), and 2021 (a3). (b) Proportional distribution of CCD categories in 2013 (b1), 2017 (b2), and 2021 (b3). (c) Trend surface analysis in 2013 (c1), 2017 (c2), and 2021 (c3).

This study visualizes the regional distribution of CCD for 2013, 2017, and 2021, as illustrated in Fig 4a. In 2013, high-value CCD areas were primarily in the eastern region, like Beijing, Guangdong, and other provinces, with Beijing being the most prominent. In contrast, low CCD values were primarily found in the west, including Ningxia, Gansu, and Guizhou. By 2017, provinces like Beijing, Guangdong, and Jiangsu in the east, as well as Hunan and Sichuan in the central and western areas, entered the high-value area for the first time. In 2021, the CCD continued the development trend of 2017, as high-value clusters persisted in the east and low-value areas remained primarily in the west.

As shown in Fig 4b, in 2013, the general out of balance category predominated, accounting for 81%, while provinces at the barely and primary coupling coordination levels constituted only 13% and 6%, respectively. Among these, the provinces at the barely coupling coordination level included Tianjin, Jiangsu, Zhejiang, and Shandong, while the only provinces at the primary coupling coordination level were Beijing and Guangdong. In 2017, the CCD level increased slightly, with the proportion of provinces at the coordinated level rising to 42%. Additionally, Anhui, Henan, Hunan, Chongqing, and Sichuan progressed from the general out-of-balance category to the barely coupling coordination level. In 2021, the CCD level rose again, with the proportion of provinces at the coordinated level increasing to 61%. Beijing, Jiangsu, Zhejiang, and Guangdong achieved the good coupling coordination level, while the western provinces of Qinghai, Ningxia, and Gansu remained at lower levels.

Trend-surface analysis was conducted using ArcGIS 10.8 software. Three-dimensional spatial perspective maps were created based on the CCD for 2013, 2017, and 2021, to reveal the overall spatial pattern and evolutionary trend of the CCD. As shown in Fig 4c, the X, Y, and Z-axis denote the west-to-east direction, the south-to-north direction, and the CCD, respectively. Throughout the study period, the CCD spatial trend generally followed a “high in the east and south, low in the west and north” pattern. The east-west trend is characterized by a “gradual increase from west to east”, with a gradual flattening of the east-west curvature of the surface. This indicates that the CCD in the eastern regions remains relatively high, while the disparity between the eastern and western areas is gradually narrowing. The CCD trend distribution along the north-south axis forms an inverted U-shape, characterized by a “raised middle section and slightly lower ends”, with the northern slope being steeper than that of the south. This indicates that the CCD in the central region exceeds that of both the southern and northern regions, with the southern region outperforming the northern region.

#### 3.2.3 The spatial agglomeration of CCD.

**Fig 5:**
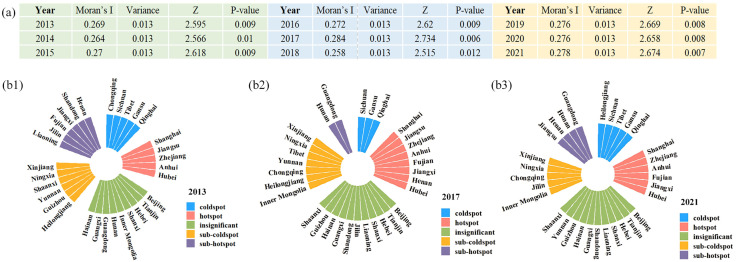
Spatial agglomeration of CCD. (a) Global Moran’s I results. (b) Distribution of cold and hot spots of CCD in 2013 (b1), 2017 (b2), and 2021(b3).

To measure the spatial dependence of CCD in China, the global Moran’s I is utilized. In Fig 5a, all CCD values pass the significance test and exhibit positive spatial autocorrelation. The CCD displays spatial clustering characteristics, indicating that the CCD of each province is influenced by neighboring provinces. Additionally, Moran’s I exhibits a fluctuating increase, suggesting an increasing trend in the spatial agglomeration of CCD values.

The global Moran’s I captures the general spatial clustering patterns of the CCD among DI, ER and CP. However, it struggles to indicate specific high-value (hotspot) or low-value (coldspot) clustering areas. To better analyze the evolution of local hotspots and coldspots, this study employs the natural breaks classification method in ArcGIS 10.8 software to categorize the CCD into five groups: hotspot, sub-hotspot, insignificant, sub-coldspot, and coldspot areas. The Getis-Ord Gi* statistic for CCD was calculated for the years 2013, 2017, and 2021, as shown in Fig 5b.

From 2013 to 2021, the spatial pattern of hotspots and coldspots in the CCD of DI, ER and CP in China exhibited significant regional disparities and dynamic shifts. Eastern regions including Jiangsu, Shanghai, Zhejiang, and Anhui consistently remained in the hotspot category, reflecting their advantages in digital infrastructure development, economic resilience, and industrial structure optimization. Since 2017, central provinces including Henan, Hubei, and Jiangxi have gradually risen into the hotspot category, demonstrating the effectiveness of national initiatives aimed at boosting Central China’s development and fostering regional balance. These regions have bolstered their digital infrastructure investment and industrial upgrading, thereby enhancing ER and significantly improving their CCD. However, western and some northeastern provinces, including Xinjiang, Gansu, Qinghai, and Jilin, remain coldspot areas. The primary reason is the imbalanced development between high-carbon-dependent industries and the digital economy, which has caused their CCD to consistently remain below the national average. Additionally, some developed provinces such as Beijing and Guangdong, despite exhibiting high CCD values, are classified as insignificant areas because of the insufficient agglomeration effect in neighboring regions. Overall, the spatial pattern of CCD reveals high levels in eastern regions, modest progress in the central areas, and comparatively lower performance in the west. Looking ahead, it will be essential to further promote regional synergistic development and strengthen policy support to narrow regional disparities and enhance overall coordination.

#### 3.2.4 Dynamics evolution trend.

In order to further analyze the probability of shifting the CCD of DI, ER and CP over time in China, this study reclassifies the CCD grades from the previous paper into four types based on their levels: low coupling coordination (Ⅰ), medium-low coupling coordination (Ⅱ), medium-high coupling coordination (Ⅲ), and high coupling coordination (Ⅳ).

**Fig 6:**
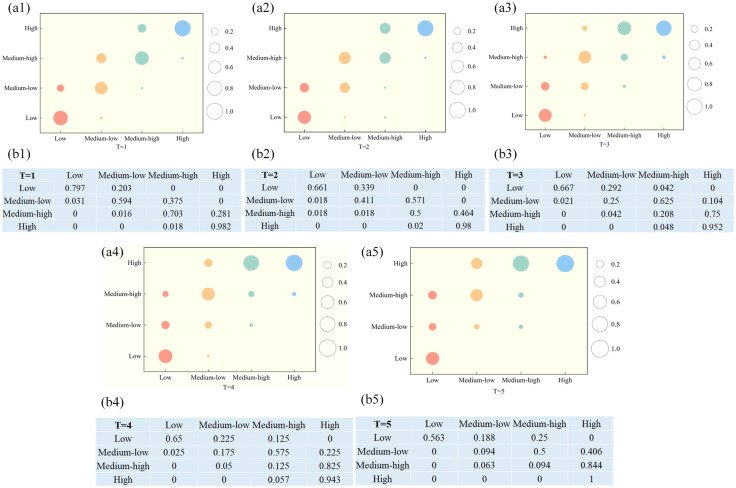
Traditional MTPX for the CCD. (a) Traditional Markov chain visualization results. (b) Traditional Markov chain results.

In the traditional Markov transition probability matrix (MTPX), the main diagonal elements represent the probability of a province’s CCD remaining stable, reflecting the consistency in its CCD evolution. The off-diagonal elements indicate the probability of transitions between different types of CCD within the province. From this matrix, we can derive the evolutionary characteristics of the CCD without considering spatial factors, as illustrated in Fig 6. The findings reveal the following: (1) With the increase in time span, the probability values along the main diagonal decrease progressively for all provinces, except those with high CCD. For instance, the values drop from P_11_ = 79.7%, P_22_ = 59.4%, and P_33_ = 70.3% at T = 1 to P_11_ = 56.3%, P_22_ = 9.4%, and P_33_ = 9.4% at T = 5, indicating significant fluctuations in the CCD of the three systems across provinces. (2) Upward transitions in CCD occur more frequently than downward shifts, indicating a stronger upward inertia. This indicates a long-term growth trend in CCD, aligning with the findings from the earlier time series analysis. Additionally, most transitions in CCD occur between adjacent levels, reflecting a relatively stable and continuous evolutionary process, making leapfrog development challenging to achieve quickly. (3) There is a potential convergence towards higher levels of coupling coordination. Provinces with high CCD maintain a stability probability of over 90% during periods 1–5, indicating that these provinces exhibit stability and self-reinforcement.

The traditional MTPX overlooks the fact that the regional CCD types can be influenced by the transitions in neighboring provinces. The coordinated development of DI, ER and CP is not spatially independent. Therefore, incorporating spatial factors is essential to explore the evolutionary traits of the CCD. This section employs the spatial Markov chain method to further reveal the spatial transfer patterns of CCD and to assess whether the CCD transitions in neighboring regions affect the CCD transitions in the focal region.

Table 4 illustrates the results of spatial factors on the dynamic evolution of CCD distribution. The findings indicate that: (1) The transition of CCD is not an isolated process; the CCD of neighboring regions impacts the transition probability of the local CCD state. Compared to the traditional MTPX, the spatial MTPX is altered when accounting for the influence of neighboring levels. Specifically, without considering the geographical spatial pattern, the probability of maintaining a primary coordination state in the traditional MTPX is 59.4% at T = 1. However, when considering the influence of neighboring development levels, the probabilities of remaining in the primary coordination state are 75%, 50%, 56%, and 33.3%, respectively, highlighting the significant impact of neighboring regions on the state transitions of the local area. Thus, spatial influences must be incorporated when analyzing the dynamics of CCD transitions. (2) The coordinated development level of DI, ER and CP exhibits a “spatial spillover” effect, where the CCD of a region is influenced by neighboring regions. When adjacent to regions with a high CCD, the probability of upward transitions increases, indicating that provinces with high CCD play a leading role in influencing surrounding provinces. For instance, at T = 1, as the CCD of neighboring regions improves, the probability of regions with a medium-low level transitioning upwards increases to 20%, 43.8%, 44%, and 66.7%, respectively, showing an upward trend. Conversely, provinces with low CCD tend to impede progress in surrounding regions. Overall, the probability of upward transitions in CCD for provinces is greater than that of downward transitions. (3) Regions with a high CCD demonstrate high transition stability and are less likely to be influenced by the CCD levels of surrounding provinces, whereas regions with low CCD have weaker stability and are more susceptible to the CCD levels of neighboring provinces.

**Table 4:** Spatial MTPX for the CCD.

Type of lag		T = 1				T = 5			
		Ⅰ	Ⅱ	Ⅲ	Ⅳ	Ⅰ	Ⅱ	Ⅲ	Ⅳ
**Low**	**Ⅰ**	0.821	0.179	0.000	0.000	0.722	0.111	0.167	0.000
	**Ⅱ**	0.050	0.750	0.200	0.000	0.000	0.222	0.667	0.111
	**Ⅲ**	0.000	0.000	0.400	0.600	0.000	0.000	0.400	0.600
	**Ⅳ**	0.000	0.000	0.000	0.000	0.000	0.000	0.000	0.000
**Medium-low**	**Ⅰ**	1.000	0.000	0.000	0.000	0.714	0.286	0.000	0.000
	**Ⅱ**	0.063	0.500	0.438	0.000	0.000	0.100	0.400	0.500
	**Ⅲ**	0.000	0.045	0.864	0.091	0.000	0.182	0.000	0.818
	**Ⅳ**	0.000	0.000	0.000	1.000	0.000	0.000	0.000	1.000
**Medium-high**	**Ⅰ**	0.333	0.667	0.000	0.000	0.000	0.000	1.000	0.000
	**Ⅱ**	0.000	0.560	0.440	0.000	0.000	0.000	0.545	0.455
	**Ⅲ**	0.000	0.000	0.583	0.417	0.000	0.000	0.167	0.833
	**Ⅳ**	0.000	0.000	0.000	1.000	0.000	0.000	0.000	1.000
**High**	**Ⅰ**	0.429	0.572	0.000	0.000	0.000	0.500	0.500	0.000
	**Ⅱ**	0.000	0.333	0.667	0.000	0.000	0.000	0.000	1.000
	**Ⅲ**	0.000	0.000	0.680	0.320	0.000	0.000	0.000	1.000
	**Ⅳ**	0.000	0.000	0.048	0.952	0.000	0.000	0.000	1.000

### 3.3 Influencing factor identification

#### 3.3.1 Influencing factor selection.

Referring to existing studies, this paper selects strategic emerging industries [45], population density [46], human capital [47], environmental regulation intensity [48], and financial development [49] as the influencing factors for CCD. Among them, strategic emerging industries (SEI) are measured by the logarithm of business income of high-tech industries. Population density (PD) is defined as the number of individuals per square kilometer. Human capital (EDU) is represented by the ratio of undergraduate students in higher education to the number of permanent residents at year-end. Environmental regulation intensity (ERI) is measured by the ratio of environmental protection expenditures to GDP. Financial development (FD) is defined as the ratio of loan balances of all financial institutions to GDP.

#### 3.3.2 Spatial model selection.

Before performing spatial econometric analyses, necessary for model specification. First, the LM and Robust LM tests were statistically significance, indicating the SDM is most appropriate. Second, the LR and Wald tests rejected the null hypothesis, confirming that the SDM cannot be simplified to either the SLM or SEM. Third, the Hausman and LR tests support the use of the SDM with two-way fixed effects. The results are in Table 5.

**Table 5:** Test results related to model selection.

Test	Statistic	Test	Statistic
LM−Error	22.416^***^	Wald− SDM/SEM	30.67^***^
LM−Lag	22.282^***^	Wald− SDM/SAR	40.97^***^
Robust LM−Error	2.904^*^	LR−both/ind	48.20^***^
Robust LM−Lag	2.771^*^	LR−both/time	887.24^***^
LR−SDM/SEM	29.56^***^	Hausman	192.19^***^
LR−SDM/SAR	37.67^***^		

Notes: ***p < 0.01, *p < 0.1.

#### 3.3.3 Benchmark regression results.

Table 6 shows a significantly positive spatial autoregressive coefficient, indicating spatial positive spillover effects in the CCD among DI, ER and CP of China. This suggests that regional CCD is shaped by both internal dynamics and external spatial influences.

**Table 6:** Benchmark regression of SDM.

Variable	Coefficient	Variable	Coefficient
*SEI*	0.0090^***^ (0.0031)	W*SEI	0.0269^***^ (0.0068)
*PD*	0.0014^***^ (0.0001)	W*PD	−0.0008^***^ (0.0002)
*EDU*	1.7383^**^ (0.7669)	W*EDU	−2.2447 (1.6014)
*ERI*	0.7401^**^ (0.3338)	W*ERI	1.8999^***^ (0.7099)
*FD*	−0.0002^**^ (0.0001)	W*FD	−0.0001 (0.0002)
*rho*	0.2873^***^ (0.0775)	*Log-likelihood*	827.0145
*sigma2_e*	0.0001^***^ (0.0000)	*R* ^*2*^	0.6942

Notes: ***p < 0.01, **p < 0.05; standard errors are reported in parentheses.

(1) SEI positively affects CCD, indicating that a larger SEI scale facilitates greater synergy among the three systems. SEI, driven by new-generation information technologies, continuously foster innovation networks and technological advancements [50], which provide essential inputs for the expansion of DI and enhancement of ER [51]. As DI expands, new technologies like 5G and AI optimize energy consumption structures [52], reduce energy consumption in enterprise production processes, and positively impact environmental performance [53]. Therefore, SEI elevates DI and ER levels, supports the advancement of a low-carbon economy, and promotes the CCD. Furthermore, the spatial lag coefficient of SEI is significantly positive, suggesting that SEI expansion in neighboring regions positively affects local CCD. The spatial agglomeration of SEI not only promotes inter-regional technology cooperation and transfer [54], but also fosters the creation and wide dissemination of new knowledge and technology [55].
(2) PD positively affects CCD, indicating that higher PD contributes to the coupling coordination among DI, ER and CP. In areas with high PD, increased resident consumption stimulates the platform economy, resulting in a continuous rise in demand for DI. Moreover, densely populated cities tend to foster agglomeration and scale effects [56]. This facilitates the effective allocation and utilization of energy, reduces the marginal cost of emission reduction, while enhancing CP. Consequently, the expansion of PD accelerates the development of DI and CP, narrows the gap with the ER system, and promotes greater coordination among the three systems. However, the spatial lag term for PD is significantly negative. This suggests that higher PD in the neighboring areas has an inhibitory effect on the local CCD. This may be due to the fact that PD is often linked to the degree of economic development, with more economically developed regions promoting a large inflow of people. The concentration of population in neighboring areas can lead to a low concentration of local industries, inefficient resource utilization, and shortages or underutilization of infrastructure, which is detrimental to the CCD locally.
(3) EDU positively impacts the CCD of DI, ER and CP, driving CCD improvement. The enhancement of EDU brings high-quality and innovative talent to DI, accelerating the progress of digital industrialization and industrial digitization, which provides a material basis for coping with unexpected crises. Additionally, the improvement of EDU contributes to raising individual environmental awareness. Broad-based support for green technology innovation and deployment facilitates clean energy substitution and environmental improvement. Although the spatial lag of EDU is negative, it does not reach a significant level. This may be because talent is the foremost resource, and the competition for talent is intensifying across regions. Regions with high EDU exhibit a “siphoning effect”, hindering the coordination of the three couplings in neighboring areas.
(4) ERI positively influences CCD, indicating that greater ERI favors the coordinated development of the three systems. Porter’s hypothesis [57] suggests that appropriate ERI compels enterprises to innovate in technology, promoting industrial restructuring [58]. On one side, this upgrading leads to a diversified industry base, which enhances the ability to cope with various risks by diversifying the demand for inputs and outputs, thereby improving ER [59]. On the other side, it accelerates digital transformation and facilitates the growth of DI such as smart transport, smart energy and smart cities. High-intensity environmental regulation enhances the developmental convergence of the three systems. Meanwhile, the markedly positive spatial lag effect of ERI indicates that the ERI in neighboring regions favorably influences local CCD development. This may be due to policy learning, imitation and mutual competition in environmental regulation between regions. Environmental regulation in one region influences the policies of neighboring regions, contributing to the improvement of CCD in those areas [60].
(5) FD negatively affects CCD, indicating that elevated FD is detrimental to the harmonized progress of the three systems. One possible reason is that financial institutions typically prefer traditional projects with low risk and high returns [61], whereas digital infrastructure and carbon productivity enhancement projects usually have high initial costs and long payback periods. Such investment preferences can constrain the financial support to emerging technologies and low-carbon economies, thus hindering the development of DI and the improvement of CP, which in turn affects ER. The spatial lag coefficient for FD is negative but not significant. This may be because the capital and talent essential for FD are highly mobile. Financial competition is prone to the “siphon effect”, where the increase in FD in one region hinders the coupling and coordination in neighboring regions.

#### 3.3.4 Direct and indirect effects.

To characterize the extent and orientation of the spatial spillovers of influencing factors, this research evaluates their direct and indirect effects [62]. Table 7 reports the results. The direct effect measures how a local indicator variable influences local CCD, while the indirect effect captures how changes in an indicator variable in neighboring regions affect local CCD. The total effect combines both these influences.

**Table 7:** Direct and indirect effects.

Variable	Total effect	Direct effect	Indirect effect
*SEI*	0.0508^***^ (0.0086)	0.0112^***^ (0.0032)	0.0396^***^ (0.0076)
*PD*	0.0008^***^ (0.0002)	0.0013^***^ (0.0001)	−0.0005^***^ (0.0002)
*EDU*	−1.0610 (1.7235)	1.6281^**^ (0.7667)	−2.6891 (1.9129)
*ERI*	3.8137^***^ (1.1397)	0.9268^**^ (0.3857)	2.8869^***^ (0.9206)
*FD*	−0.0005 (0.0004)	−0.0003^**^ (0.0001)	−0.0002 (0.0003)

The direct and indirect effects of SEI and ERI are notably positive, showing that SEI and ERI development promotes the CCD both locally and in neighboring regions. The direct impact of PD is markedly positive, while the indirect impact is markedly negative. However, the total effect remains positive, suggesting that the beneficial influence of PD on the CCD primarily originates from the local area. The direct impact of EDU is significantly positive, while the indirect impact is insignificant, suggesting that improvements in EDU promote CCD development locally, though the coupling effect on neighboring areas is not evident. The direct effect of FD is significantly negative, while its indirect and total effects are insignificant, implying that the current level of FD does not improve the CCD.

## 4 Discussion

### 4.1 Subsystem analysis

The comprehensive indices of the three subsystems showed an overall increasing trend, as illustrated in Fig 2a and b. Bai et al. [63] highlighted that CP has exhibited a year-on-year upward trajectory, primarily driven by technological progress. Furthermore, DI development and environmental regulation have been identified as important factors contributing to CP enhancement [37,64]. The sustained rise in DI levels reflects the growing demand induced by advances in information technology and the internet [65]. The rising trend of ER is consistent with the findings of Song et al. [66]. Policy initiatives, such as the New Energy Demonstration City Policy, have promoted regional ER by alleviating financing constraints and optimizing capital structures [67]. However, significant regional disparities persist in the advancement of DI, ER and CP. In particular, DI in the eastern region advanced earlier and more rapidly [68], fueled by intensive investments in frontier technologies including 5G, the industrial internet, and AI. The agglomeration of capital and technology further reinforced the expansion of DI, which in turn accelerated improvements in ER and CP. In contrast, the western region continues to lag behind in both ER and CP, a finding that aligns with previous studies [69,70]. This disparity is primarily attributable to variations in industrial structure. The eastern and central areas are predominantly characterized by service industries and technology-intensive manufacturing. Industrial diversification not only enhances economic self-sufficiency and resilience against external shocks [71], but also creates favorable conditions for stringent environmental regulations, thereby facilitating cleaner energy transitions and improving CP [72,73]. Similarly, Li and Wang [70] observed that CP in eastern provinces markedly exceeds that of the central and western areas. They further emphasized that a higher share of traditional industrial activities impedes improvements in CP, whereas technological innovation combined with managerial efficiency fosters its growth.

### 4.2 Coupling coordination analysis

Fig 3a shows a high level of CD, indicating strong interlinkages among the three systems. Fig 3b illustrates that the CCD exhibits an overall rising trajectory with fluctuations, with the differences among the three systems gradually narrowing and converging in terms of state and development speed. The coupling status has improved from “general out of balance” to “barely coupling coordination”. At the subsystem level, although ER maintains the highest overall level, CP grows fastest, followed by DI. This contrasts with Fan and Li [74], who found ecological protection leading in development level and the digital economy growing most rapidly. In this study, the rapid catch-up momentum of CP and DI narrowed their gap with ER, thereby driving improvements in CCD. In particular, 2015 marked the sharpest increase in CCD, likely due to simultaneous advancements in national digital and environmental policies. On the one hand, Zhang et al. [75] pointed out that during this period, China proposed and promoted the “Internet Plus” action plan, accelerating the convergence of digital technologies and traditional industries, thereby promoting DI. The development of DI contributes to the reduction of carbon emission intensity. Relevant studies have confirmed that a 1% increase in DI leads to a 0.019% reduction in carbon intensity [8]. On the other hand, Zhang and Cao [76] describe the amended Environmental Protection Law of 2015 as the most stringent in China’s history, and Yu and Morotomi [77] confirm that its implementation significantly reduced urban pollution through enhanced enforcement. Although the growth rate of ER slowed after 2019, it still maintained the highest level among the subsystems. This stagnation may be attributed to the disruptions caused by COVID-19, during which DI played a key role in mitigating its adverse impact on ER, as evidenced by Wen et al. [78] and Zou [79]. A 1% increase in DI results in a 0.0083% improvement in ER by enabling better coordination between the digital and real economies during the pandemic [78]. According to coupling coordination theory, the synergistic development of DI, ER, and CP strengthens inter-system interactions, thereby enhancing overall system coordination. Therefore, future efforts should focus on integrating digital and low-carbon transitions while reinforcing ER, in order to promote higher CCD and advance sustainable urban development.

Fig 4a–c illustrate the CCD results for each province, revealing a general upward trajectory across most provinces and a clear spatial gradient—high in the east and south, and low in the west and north. Only four eastern provinces have reached an intermediate level of coordination. This aligns with existing research regarding the connection between DI and ecological environment [11,47]. The reason for this disparity is the imbalance in digitalization [80] and ecological protection [81] in different provinces of China. The eastern region benefits from both its strategic location and abundant resources, and it primarily focuses on industries that are high value-added, environmentally friendly, and technology-driven. When the government proposes to build DI, it can respond quickly with capital, talent, and technology, achieving positive interaction among the three systems more rapidly. Most provinces in the central region have shifted from dysfunctional to coordinated, with improvements in the coupling of DI, ER and CP. Notably, digitalization is essential to enhancing the CCD. By fostering green innovation and promoting environmental investment, digitalization effectively drives corporate emission reductions and improvements in environmental performance. Particularly under the influence of environmentally conscious leadership, firms are more inclined to integrate environmental protection measures into their digitalization processes [82]. This mechanism is most pronounced in the eastern and central regions, where stringent environmental regulations and green innovation have significantly enhanced CP, thereby further promoting the CCD. However, in western areas, due to lagging infrastructure development and insufficient levels of digitalization, their role in enhancing the CCD of the three systems is significantly constrained. Currently, most provinces in western China remain in a state of maladjusted development. This observation is consistent with prior studies on the interactions among social, economic, and environmental systems [83,84]. The western region faces practical challenges, including sparse populations, lower levels of economic development, and an insufficient scale of SEI [85]. These factors limit the region’s capacity to accommodate the development of DI. The lag in DI construction further hinders the synergistic enhancement of ER and CP, making it difficult to establish a virtuous cycle of interaction among the three systems. Therefore, reducing regional disparities in low-carbon development and addressing the digital divide are the urgent priorities for promoting balanced national development.

## 5 Conclusions

This paper uses a sample of 31 provinces in China to establish an index system for DI, ER and CP. This study applies the CCD model, spatial autocorrelation, trend-surface analysis, and SDM to examine the spatiotemporal evolution of CCD and its driving factors. The findings indicate that:

First, the levels of DI, ER and CP all increased steadily over the period from 2013 to 2021. During this period, the average annual growth rates of DI, ER, and CP were 6.88%, 5.92%, and 7.55%, respectively. The eastern region maintained a consistent lead, with the annualized growth rates of DI, ER, and CP were 7.35%, 5.79%, and 6.67%, respectively. Although the central and western regions experienced improvement, they remained below national averages. Notably, the western region overtook the central region in CP after 2017, while its earlier advantage in ER was reversed after 2015.

Second, the average CCD exhibited a fluctuating yet overall upward trend over time, rising from 0.331 in 2013 to 0.430 in 2021, representing a growth of 29.91%. Except for Liaoning Province, where the CCD declined slightly by 0.59%, all other provinces showed steady annual increases. Spatially, CCD demonstrated a distinct pattern, described as “high in the east and south, low in the west and north”, with Beijing, Guangdong, and Jiangsu provinces achieving the highest CCD of 0.674, 0.635, and 0.546, respectively, while Ningxia, Gansu, and Qinghai provinces reported the lowest values of 0.252, 0.255, and 0.276. Overall, achieving a “cross-level transition” in CCD is challenging, and neighboring provinces exert a significant influence on the enhancement of local CCD.

Third, CCD exhibited significant positive spatial clustering characteristics. During the study period, the Global Moran’s I rose from 0.269 to 0.278, confirming positive spatial autocorrelation. Stable hotspots were consistently identified in Hubei, Anhui, Zhejiang, and Shanghai provinces, whereas stable coldspots were mainly concentrated in Gansu, Qinghai, and Sichuan. Overall, the spatial distribution of CCD followed a consistent pattern, being stronger in the east, weaker in the west, and showing gradual improvement in the central areas.

Fourth, a significant spatial correlation was observed among the CCD of DI, ER and CP. Spatial econometric analysis revealed that the strategic emerging industries and environmental regulation intensity both exerted positive spatial spillover effects (0.0269 and 1.8999). Population density exhibited a negative spatial spillover effect (−0.0008). Human capital positively affected local CCD but lacked significant spatial spillover effects, while financial development exhibited no significant spatial influence. These results underscore the critical roles of economic vitality and education in enhancing regional CCD performance.

## 6 Policy implications

Drawing upon the empirical results, the following policy implications are proposed to strengthen the CCD among DI, ER and CP:

First, given the steady improvements in DI, ER, and CP but persistent regional disparities, differentiated policy frameworks should be implemented to reinforce growth momentum. Eastern China should focus on integrating digital technologies with green manufacturing, energy, and infrastructure systems. In the central and western areas, governments should increase investment in broadband networks and smart infrastructure. Efforts should also accelerate the digital transformation of conventional sectors and incentivize the deployment of low-carbon technologies via subsidy schemes. In addition, initiatives are required to sustain the CP gains in the western areas, including the establishment of technology transfer centers, the creation of green finance platforms, and the construction of technology parks. These measures are critical for enhancing the spatial coordination of DI, ER, and CP and for fostering sustainable regional development.

Second, recognizing the significant heterogeneity in CCD across provinces and developmental stages, policymakers should prioritize region-specific development strategies tailored to local strengths and weaknesses. Efforts should focus on consolidating the eastern region’s advantages in DI, ER and CP. Concurrently, it is essential to enhance the development capacity of the central and western provinces through strengthened institutional support, improved technological capabilities, and the creation of enabling environments for low-carbon and digital transitions. To promote spatially balanced development, greater emphasis should be placed on facilitating the flow of capital, human resources, and technology from economically advanced provinces to underdeveloped regions, thereby reinforcing the coordinated integration of DI, ER, and CP.

Third, national and provincial authorities should establish dynamic monitoring systems based on CCD to track regional development in real time and inform policy decisions. Monitoring outcomes should be utilized to identify fluctuations in spatial relationships and enable timely policy adjustments. In parallel, efforts should be directed toward fostering cross-regional collaborative innovation platforms to enhance spatial spillover effects. These may include joint research centers, talent mobility programs, and coordinated industrial parks connecting leading provinces such as Beijing, Guangdong, and Zhejiang with less-developed regions like Gansu and Qinghai. Policy support should be provided through dedicated interregional collaboration funds, preferential tax policies for cross-regional innovation activities, and investment in shared technological infrastructure. Leveraging the technological and financial strengths of eastern provinces can promote resource redistribution and facilitate more balanced regional development, thereby reinforcing the integrated advancement of DI, ER, and CP.

Fourth, given the significant spatial correlation in the CCD of DI, ER and CP, policy interventions should aim to enhance positive spatial spillovers and mitigate negative ones. National and local governments should prioritize the cultivation of strategic emerging industries through fiscal incentives, innovation subsidies, and cross-regional industrial alliances. Environmental regulations should be adapted to local conditions while maintaining stringency to stimulate green investment. Urban planning strategies should guide rational population distribution by supporting the development of medium-sized cities and reducing excessive concentration in core urban areas. Additionally, interregional talent exchange platforms and joint training initiatives should be established to improve the spatial mobility of human capital. Financial development should be oriented toward improving interregional capital flow through inclusive financing tools and regional investment coordination mechanisms.

## Supporting information

S1 Dataset Raw data of the subsystem. (XLSX)

S2 Dataset Carbon productivity results. (XLSX)

S3 Dataset Digital infrastructure results. (XLSX)

S4 Dataset Economic resilience results. (XLSX)

S5 Dataset Coupling coordination degree results. (XLSX)

S6 Dataset Coupling degree results. (XLSX)

S7 Dataset Raw data of influencing factors. (XLSX)
